# The Daughter

**Published:** 1891-05

**Authors:** 


THE DAUGHTER: HER HEALTH, EDUCATION, AND WEDLOCK. Homely Suggestions for Mothers and Daughters. By William M. Capp, M. D. Philadelphia: 1231 Filbert Street, F. A. Davis, Publisher. 1891. Price, $1.00 net.

This elegant little volume should be in the hands of every mother having a daughter to raise. It gives plain and simple directions for the management of every event in the life of a daughter from birth to marriage. It is divided into short paragraphs, no chapters, each paragraph being a concise and clear statement for the guidance of the mother. At the end of the book is an index by which any paragraph may be readily found. It is printed in large, clear type, and is tastefully bound. We cordially recommend it. W. M. J.

